# Crystal structure of (*RS*)-(4-chloro­phen­yl)(pyridin-2-yl)methanol

**DOI:** 10.1107/S2056989015023154

**Published:** 2016-01-01

**Authors:** Badiadka Narayana, Balladka K. Sarojini, Hemmige S. Yathirajan, Ravindranath Rathore, Christopher Glidewell

**Affiliations:** a Department of Studies in Chemistry, Mangalore University, Mangalagangothri 574 199, India; b Department of Studies in Industrial Chemistry, Mangalore University, Mangalagangothri 574 199, India; c Department of Studies in Chemistry, University of Mysore, Manasagangotri, Mysuru 570 006, India; d Department of Biotechnology, Dayananda Sagar College of Engineering, Bengaluru 560 078, India; e School of Chemistry, University of St Andrews, Fife KY16 9ST, Scotland

**Keywords:** crystal structure, supra­molecular structure, hydrogen bonds, halogen–pyridine inter­actions

## Abstract

In the title racemic compound, C_12_H_10_ClNO, the dihedral angle between the benzene and pyridine rings is 74.34 (6)°. In the crystal, the mol­ecules are linked by O—H⋯N hydrogen bonds, forming zigzag *C*(5) [001] chains in which alternating *R*- and *S*-configuration mol­ecules are related by *c*-glide symmetry. In addition, inversion-related pairs of mol­ecules are linked into dimers by pairs of weak C—Cl⋯π(pyrid­yl) inter­actions, which link the hydrogen-bonded chains into (100) sheets. Structural comparisons are drawn with a number of related compounds.

## Abstract 2

A combination of O—H⋯N hydrogen bonds and C—Cl⋯π(pyrid­yl) inter­actions links the mol­ecules of the title compounds into (100) sheets.

## Chemical context   

**Figure 1:**
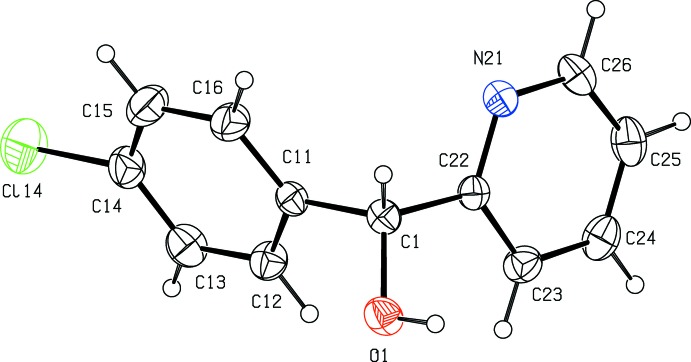
The mol­ecular structure of the *R*-enanti­omer of compound (I), showing the atom-labelling scheme. Displacement ellipsoids are drawn at the 30% probability level.

Simply substituted di­phenyl­methanols, *R*Ph_2_COH, exhibit a very rich diversity of supra­molecular arrangements, including isolated mol­ecules, hydrogen-bonded dimers, trimers, tetra­mers and hexa­mers, as well as continuous hydrogen-bonded chains (Ferguson *et al.*, 1992 ▸, 1994 ▸, 1995 ▸). The predominant mode of mol­ecular association in these structures involves O—H⋯O hydrogen bonds, although O—H⋯π(arene) inter­actions are sometimes present. It is therefore of considerable inter­est to investigate the influence of an addition potential acceptor of hydrogen bonds as achieved, for example, by the replacement of one of the phenyl rings by an isosteric pyridyl substituent. Here we report the mol­ecular and supra­molecular structure of (*RS*)-4-chloro­phen­yl(pyridin-2-yl)methanol (I) (Fig. 1 ▸), which shows some striking structural differences from the simpler, non-chlorinated analogue phen­yl(pyridin-2-yl)methanol, whose structure has been reported recently (Kim & Kang, 2014 ▸; Tsang *et al.*, 2015 ▸).
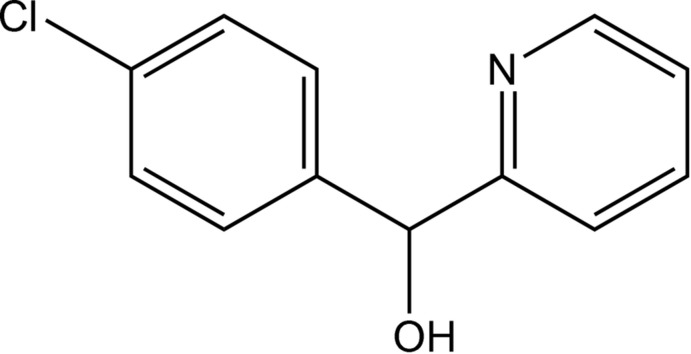


## Structural commentary   

The mol­ecules of compound (I) contain a stereogenic centre at atom C1 (Fig. 1 ▸) and the reference mol­ecule was selected as one having the *R*-configuration at atom C1. The centrosymmetric space group confirms that compound (I) has crystallized as a racemic mixture.

Both of the rings are rotated out of the plane of the central C11–C1–C22 fragment, which makes dihedral angles of 70.69 (2) and 84.66 (9)° with the phenyl and pyridyl rings, respectively. The dihedral angle between the rings is 74.34 (6)°, and this value is very similar to the value of 71.42 (10)° reported (Kim & Kang, 2014 ▸) for the corres­ponding angle in the non-chlorinated analogue, compound (II). The general conformational similarity between the mol­ecules of compounds (I) and (II) is shown by the torsional angles O—C—C—C and O—C—C—N (Table 1 ▸), where the corresponding angles for the *R*-enanti­omer of (II) [the reference mol­ecule was actually selected (Kim & Kang, 2014 ▸) as one having the *S*-configuration] are 49.0 (4) and −150.6 (2)°, respectively.

However, one point of difference between the conformations in compounds (I) and (II) centres on the locations of the hydroxyl H atoms. In compound (I), this atom is anti­periplanar to atom C11 (Table 1 ▸), but the corresponding torsional angle for the *R*-enanti­omer of (II) is −67 (2)°. This difference in hydroxyl group conformations is probably associated with the different patterns of hydrogen-bonded supra­molecular aggregation in compounds (I) and (II), as discussed below.

## Supra­molecular inter­actions   

**Figure 2:**
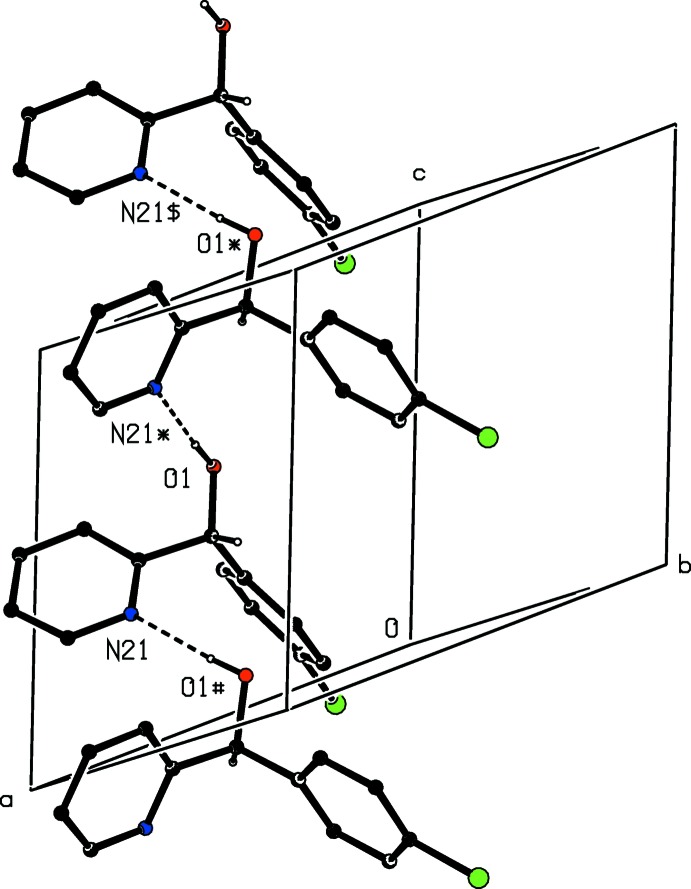
Part of the crystal structure of compound (I), showing the formation of a hydrogen-bonded *C*(5) chain containing alternating enanti­omers and running parallel to [001]. For the sake of clarity, the H atoms bonded to the ring C atoms have been omitted. The atoms marked with an asterisk (*), a hash (#) or a dollar sign ($) are at the symmetry positions (*x*, 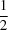 − *y*, 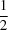 + *z*), (*x*, 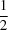 − *y*, −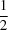 + *z*) and (*x*, *y*, 1 + *z*), respectively.

The mol­ecules of compound (I) are linked by O—H⋯N hydrogen bonds (Table 2 ▸), forming zigzag *C*(5) chains running parallel to the [001] direction. The chain containing the reference mol­ecule at (*x*, *y*, *z*) consists of mol­ecules which are related by the *c*-glide plane at *y* = 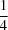, so that mol­ecules of *R*-configuration and *S*-configuration alternate along the chain (Fig. 2 ▸). Two chains of this type, related to one another by inversion, pass through each unit cell.

**Figure 3:**
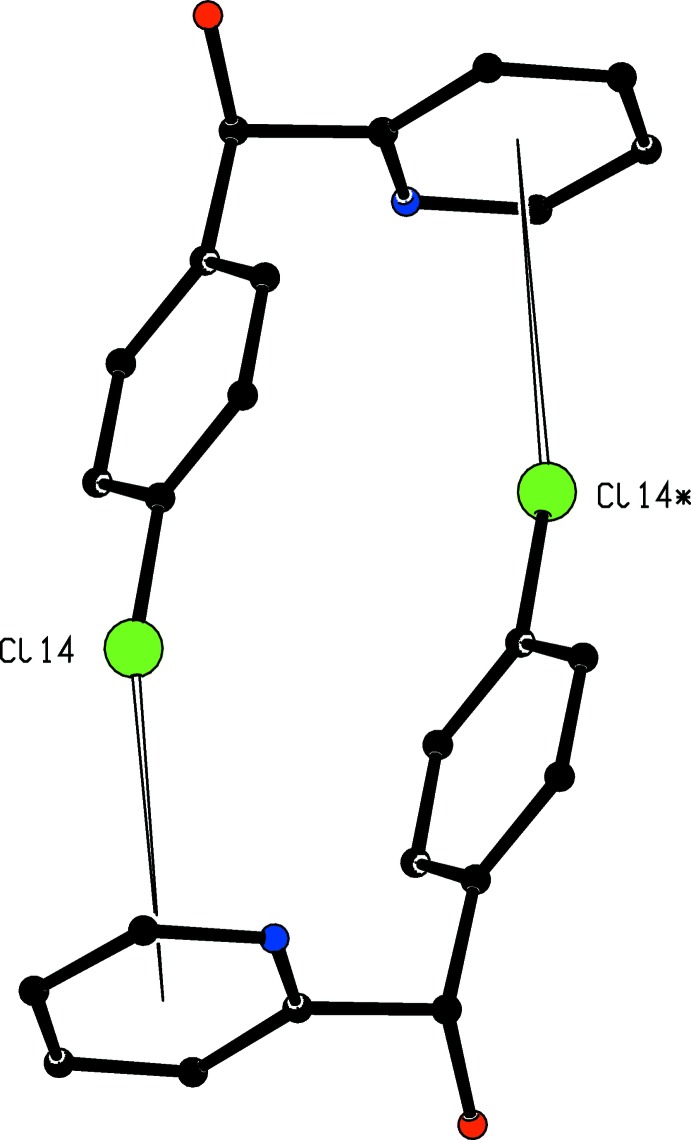
A centrosymmetric dimer in in the crystal of (I) in which the mol­ecules are linked by C—Cl⋯π inter­actions, shown as hollow lines. For the sake of clarity, all of the H atoms have been omitted. The Cl atom marked with an asterisk (*) is at the symmetry position (1 − *x*, −*y*, −*z*).

The crystal structure of compound (I) contains neither C—H⋯π hydrogen bonds nor π–π stacking inter­actions. There is, however, a single short C—Cl⋯π contact with geometric parameters Cl⋯*Cg* ^i^ = 3.5280 (10) Å, C⋯*Cg* ^i^ = 5.1785 (19) Å and C—Cl⋯*Cg* ^i^ = 157.79 (7)° [symmetry code: (i) 1 − *x*, −*y*, −*z*] where *Cg* represents the centroid of the pyridine ring. This Cl⋯*Cg* distance is slightly shorter than the average distance, 3.6 Å, deduced (Imai *et al.*, 2008 ▸) from database analysis in a study which concluded that such inter­actions were attractive, with inter­action energies of *ca* 2 kcal mol^−1^, comparable to those typical of weak hydrogen bonds (Desiraju & Steiner, 1999 ▸). In compound (I), this inter­action links inversion-related pairs of mol­ecules into cyclic centrosymmetric dimers (Fig. 3 ▸).

**Figure 4:**
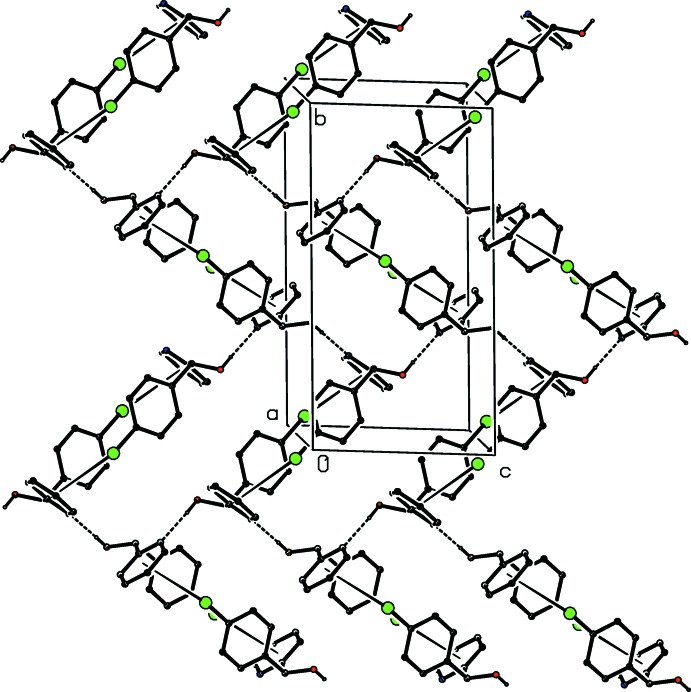
A view of part of the crystal structure of (I), showing the formation of a sheet parallel to (001) built from hydrogen-bonded chains linked by C—Cl⋯π inter­actions. For the sake of clarity, the H atoms bonded to C atoms have all been omitted.

The overall effect of the C—Cl⋯π inter­action in (I) is to link the hydrogen-bonded chain containing mol­ecules related by the *c*-glide plane at *y* = 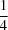 directly to the two chains that contain mol­ecules related by the glide planes at *y* = −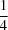 and *y* = 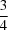, respectively, and propagation by translation of this inter­action links the hydrogen-bonded chains along [001] into a sheet lying parallel to (100) (Fig. 4 ▸), but there are no direction-specific inter­actions between adjacent sheets.

## Structural comparisons with related compounds   

**Figure 5:**
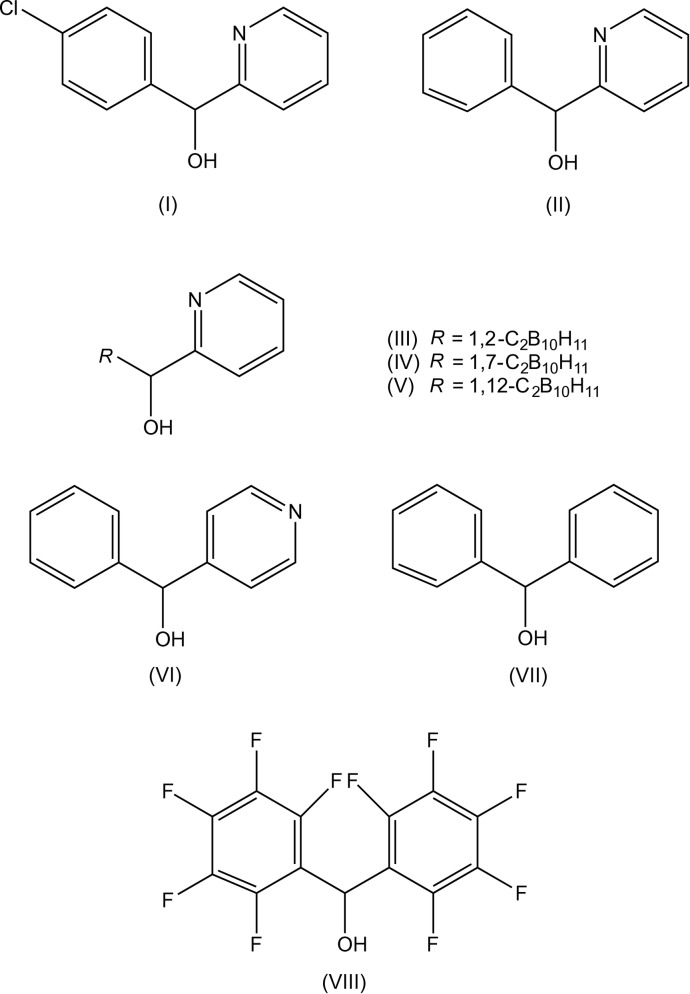
Related compounds.

It is of inter­est briefly to compare the supra­molecular assembly in compound (I), mediated by O—H⋯N hydrogen bonds and C—Cl⋯π inter­actions, with the assembly in some closely related compounds (II)–(VIII) (see Fig. 5 ▸), and particularly with compound (II), whose constitution differs from that of (I) only in lacking the chloro substituent.

The mol­ecules of compound (II) are linked into *C*(5) chains by O—H⋯N hydrogen bonds (Kim & Kang, 2014 ▸; Tsang *et al.*, 2015 ▸), as in compound (I), but in (II) helical chains are built from mol­ecules related by 2_1_ screw axes in space group *Pna*2_1_, whereas in (I) zigzag chains are built from mol­ecules related by glide planes. Hence in compound (II) each chain is homochiral, with equal numbers of chains built only from mol­ecules having the *R*-configuration or only from mol­ecules having the *S*-configuration: in (I), by contrast, each chain contains an alternation of the two enanti­omers (*cf.* Fig. 2 ▸).

Similar homochiral *C*(5) chains are formed in each of the three isomeric carborane derivatives (III)–(V) (Tsang *et al.*, 2015 ▸), regardless of whether they are crystallized as single enanti­omers or as racemates. The structure of compound (VI), which is isomeric with (II) has been reported briefly (Shimada *et al.*, 2003 ▸) but, unfortunately, no atomic coordinates have been deposited in the Cambridge Structural Database (Groom & Allen, 2014 ▸). The structure report on (VI) concerns enanti­omerically pure forms, in space group *P*2_1_2_1_2_1_, so that the formation of homochiral helical chains of *C*(7) type, seems plausible.

Compound (VII), which differs from (I) and (II) in containing two unsubstituted phenyl rings but no pyridyl ring, crystallizes with *Z*′ = 2 in space group *P*22_1_2_1_ (Ferguson *et al.*, 1995 ▸) and the mol­ecules are linked by O—H⋯O hydrogen bonds to form 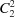
(4) chains, but with no direction-specific inter­actions between adjacent chains. Compound (VIII) is the penta­fluoro­phenyl analogue of (VII) and the mol­ecules are again linked by O—H⋯O hydrogen bonds, but now forming cyclic 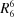
(12) hexa­mers having exact 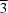 (*S*_6_) symmetry (Ferguson *et al.*, 1995 ▸).

## Synthesis and crystallization   

A sample of the title compound (I) was a gift from CAD Pharma, Bengaluru, India. Colourless blocks were grown by slow evaporation at room temperature of a solution in methanol, m.p. 478 K.

## Refinement   

Crystal data, data collection and structure refinement details are summarized in Table 3 ▸. All H atoms were located in difference maps. The H atoms bonded to C atoms were then treated as riding atoms in geometrically idealized position with C—H distances of 0.93 Å (aromatic and heteroaromatic) or 0.98 Å (aliphatic CH) and with *U*_iso_(H) = 1.2*U*_eq_(C). For the hydroxyl H atom H1*A*, the atomic coordinates were refined with *U*_iso_(H) = 1.5*U*_eq_(O), giving an O—H distance of 0.84 (2) Å. The analysis of variance reported a large value of K, 3.187, for the group of 252 very weak reflections having *F*_c_/*F*_c_(max) in the range 0.000 < *F*_c_/*F*_c_(max) < 0.005.

## Supplementary Material

Crystal structure: contains datablock(s) global, I. DOI: 10.1107/S2056989015023154/hb7553sup1.cif

Structure factors: contains datablock(s) I. DOI: 10.1107/S2056989015023154/hb7553Isup2.hkl

Supporting information file. DOI: 10.1107/S2056989015023154/hb7553Isup3.cml

CCDC reference: 1440028

Additional supporting information:  crystallographic information; 3D view; checkCIF report

## Figures and Tables

**Table 1:** Selected torsion angles (°)

O1—C1—C11—C12	−51.14 (17)	C11—C1—O1—H1*A*	−180.0 (17)
O1—C1—C22—N21	−156.41 (13)		

**Table 2:** Hydrogen-bond geometry (Å, °)

*D*—H⋯*A*	*D*—H	H⋯*A*	*D*⋯*A*	*D*—H⋯*A*
O1—H1*A*⋯N21^i^	0.84 (2)	2.01 (2)	2.8444 (18)	176 (2)

Symmetry code: (i) 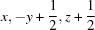.

**Table 3:** Experimental details

Crystal data	
Chemical formula	C_12_H_10_ClNO
*M*_r_	219.66
Crystal system, space group	Monoclinic, *P*2_1_/*c*
Temperature (K)	295
*a*, *b*, *c* (Å)	8.4309 (6), 16.1488 (11), 8.6878 (6)
β (°)	112.994 (2)
*V* (Å^3^)	1088.85 (13)
*Z*	4
Radiation type	Mo *K*α
μ (mm^−1^)	0.32
Crystal size (mm)	0.40 × 0.30 × 0.20

Data collection	
Diffractometer	Bruker APEXII CCD
Absorption correction	Multi-scan (*SADABS*; Sheldrick, 2003 ▸)
*T*_min_, *T*_max_	0.719, 0.938
No. of measured, independent and observed [*I* > 2σ(*I*)] reflections	11481, 2510, 1860
*R*_int_	0.030
(sin θ/λ)_max_ (Å^−1^)	0.651

Refinement	
*R*[*F* ^2^ > 2σ(*F* ^2^)], *wR*(*F* ^2^), *S*	0.045, 0.118, 1.06
No. of reflections	2510
No. of parameters	139
H-atom treatment	H atoms treated by a mixture of independent and constrained refinement
Δρ_max_, Δρ_min_ (e Å^−3^)	0.21, −0.39

Computer programs: *APEX2* and *SAINT-Plus* (Bruker, 2012 ▸), *SHELXS97* (Sheldrick, 2008 ▸), *SHELXL2014* (Sheldrick, 2015 ▸) and *PLATON* (Spek, 2009 ▸).

## supplementary crystallographic information

### Crystal data

**Table:**

C_12_H_10_ClNO	*F*(000) = 456
*M**_r_* = 219.66	*D*_x_ = 1.340 Mg m^−^^3^
Monoclinic, *P*2_1_/*c*	Mo *K*α radiation, λ = 0.71073 Å
*a* = 8.4309 (6) Å	Cell parameters from 2785 reflections
*b* = 16.1488 (11) Å	θ = 2.5–28.6°
*c* = 8.6878 (6) Å	µ = 0.32 mm^−^^1^
β = 112.994 (2)°	*T* = 295 K
*V* = 1088.85 (13) Å^3^	Block, colourless
*Z* = 4	0.40 × 0.30 × 0.20 mm

### Data collection

**Table:**

Bruker APEXII CCD diffractometer	2510 independent reflections
Radiation source: fine-focus sealed tube	1860 reflections with *I* > 2σ(*I*)
Graphite monochromator	*R*_int_ = 0.030
φ and ω scans	θ_max_ = 27.6°, θ_min_ = 2.5°
Absorption correction: multi-scan (*SADABS*; Sheldrick, 2003)	*h* = −9→10
*T*_min_ = 0.719, *T*_max_ = 0.938	*k* = −21→15
11481 measured reflections	*l* = −11→11

### Refinement

**Table:**

Refinement on *F*^2^	0 restraints
Least-squares matrix: full	Hydrogen site location: mixed
*R*[*F*^2^ > 2σ(*F*^2^)] = 0.045	H atoms treated by a mixture of independent and constrained refinement
*wR*(*F*^2^) = 0.118	*w* = 1/[σ^2^(*F*_o_^2^) + (0.0513*P*)^2^ + 0.2352*P*] where *P* = (*F*_o_^2^ + 2*F*_c_^2^)/3
*S* = 1.06	(Δ/σ)_max_ < 0.001
2510 reflections	Δρ_max_ = 0.21 e Å^−^^3^
139 parameters	Δρ_min_ = −0.39 e Å^−^^3^

### Special details

**Table:**

Geometry. All e.s.d.'s (except the e.s.d. in the dihedral angle between two l.s. planes) are estimated using the full covariance matrix. The cell e.s.d.'s are taken into account individually in the estimation of e.s.d.'s in distances, angles and torsion angles; correlations between e.s.d.'s in cell parameters are only used when they are defined by crystal symmetry. An approximate (isotropic) treatment of cell e.s.d.'s is used for estimating e.s.d.'s involving l.s. planes.

### Fractional atomic coordinates and isotropic or equivalent isotropic displacement parameters (Å^2^)

**Table:**

	*x*	*y*	*z*	*U*_iso_*/*U*_eq_	
C1	0.66425 (18)	0.19218 (9)	0.43123 (18)	0.0367 (3)	
H1	0.6340	0.2499	0.3976	0.044*	
O1	0.65282 (15)	0.17831 (8)	0.58744 (14)	0.0475 (3)	
H1A	0.725 (3)	0.2105 (14)	0.655 (3)	0.071*	
C11	0.53625 (18)	0.13554 (9)	0.30436 (19)	0.0358 (3)	
C12	0.5356 (2)	0.05176 (10)	0.3383 (2)	0.0458 (4)	
H12	0.6128	0.0313	0.4400	0.055*	
C13	0.4232 (2)	−0.00168 (11)	0.2247 (2)	0.0533 (5)	
H13	0.4239	−0.0578	0.2492	0.064*	
C14	0.3099 (2)	0.02875 (12)	0.0747 (2)	0.0548 (5)	
Cl14	0.16964 (9)	−0.03856 (4)	−0.07147 (8)	0.0922 (3)	
C15	0.3058 (3)	0.11173 (14)	0.0391 (2)	0.0668 (6)	
H15	0.2270	0.1321	−0.0619	0.080*	
C16	0.4198 (2)	0.16462 (11)	0.1546 (2)	0.0538 (5)	
H16	0.4175	0.2208	0.1304	0.065*	
N21	0.88793 (16)	0.21277 (8)	0.32519 (16)	0.0414 (3)	
C22	0.84457 (18)	0.17470 (9)	0.43935 (18)	0.0339 (3)	
C23	0.9548 (2)	0.12092 (11)	0.5561 (2)	0.0461 (4)	
H23	0.9219	0.0959	0.6353	0.055*	
C24	1.1140 (2)	0.10498 (12)	0.5534 (2)	0.0537 (5)	
H24	1.1899	0.0688	0.6304	0.064*	
C25	1.1589 (2)	0.14314 (12)	0.4359 (3)	0.0570 (5)	
H25	1.2658	0.1335	0.4314	0.068*	
C26	1.0431 (2)	0.19599 (11)	0.3247 (2)	0.0523 (4)	
H26	1.0741	0.2216	0.2446	0.063*	

### Atomic displacement parameters (Å^2^)

**Table:**

	*U*^11^	*U*^22^	*U*^33^	*U*^12^	*U*^13^	*U*^23^
C1	0.0359 (8)	0.0344 (8)	0.0421 (8)	0.0029 (6)	0.0178 (7)	0.0000 (6)
O1	0.0520 (7)	0.0526 (7)	0.0455 (7)	−0.0078 (6)	0.0272 (6)	−0.0106 (5)
C11	0.0323 (7)	0.0373 (8)	0.0411 (8)	0.0010 (6)	0.0177 (6)	−0.0001 (6)
C12	0.0452 (9)	0.0401 (9)	0.0485 (9)	0.0034 (7)	0.0142 (7)	0.0020 (7)
C13	0.0589 (11)	0.0406 (9)	0.0629 (12)	−0.0057 (9)	0.0265 (10)	−0.0055 (8)
C14	0.0524 (10)	0.0617 (12)	0.0497 (10)	−0.0168 (9)	0.0194 (8)	−0.0134 (9)
Cl14	0.0971 (5)	0.0942 (5)	0.0724 (4)	−0.0416 (4)	0.0190 (3)	−0.0316 (3)
C15	0.0667 (12)	0.0685 (13)	0.0465 (10)	−0.0118 (11)	0.0019 (9)	0.0068 (10)
C16	0.0554 (10)	0.0464 (10)	0.0502 (10)	−0.0044 (8)	0.0105 (8)	0.0095 (8)
N21	0.0390 (7)	0.0417 (7)	0.0450 (8)	−0.0018 (6)	0.0182 (6)	0.0005 (6)
C22	0.0341 (7)	0.0318 (7)	0.0351 (8)	−0.0016 (6)	0.0128 (6)	−0.0041 (6)
C23	0.0452 (9)	0.0492 (9)	0.0430 (9)	0.0064 (8)	0.0162 (7)	0.0031 (8)
C24	0.0418 (9)	0.0553 (11)	0.0563 (11)	0.0135 (8)	0.0108 (8)	−0.0005 (9)
C25	0.0357 (8)	0.0633 (12)	0.0748 (13)	0.0032 (9)	0.0245 (9)	−0.0093 (10)
C26	0.0459 (9)	0.0566 (11)	0.0637 (11)	−0.0050 (9)	0.0315 (9)	−0.0009 (9)

### Geometric parameters (Å, º)

**Table:**

C1—O1	1.4154 (18)	C15—C16	1.381 (3)
C1—C11	1.512 (2)	C15—H15	0.9300
C1—C22	1.5206 (19)	C16—H16	0.9300
C1—H1	0.9800	N21—C22	1.3335 (19)
O1—H1A	0.84 (2)	N21—C26	1.338 (2)
C11—C16	1.371 (2)	C22—C23	1.382 (2)
C11—C12	1.385 (2)	C23—C24	1.376 (2)
C12—C13	1.373 (2)	C23—H23	0.9300
C12—H12	0.9300	C24—C25	1.367 (3)
C13—C14	1.371 (3)	C24—H24	0.9300
C13—H13	0.9300	C25—C26	1.370 (3)
C14—C15	1.373 (3)	C25—H25	0.9300
C14—Cl14	1.7376 (18)	C26—H26	0.9300
			
O1—C1—C11	107.86 (12)	C16—C15—H15	120.3
O1—C1—C22	111.56 (12)	C11—C16—C15	121.00 (17)
C11—C1—C22	109.72 (11)	C11—C16—H16	119.5
O1—C1—H1	109.2	C15—C16—H16	119.5
C11—C1—H1	109.2	C22—N21—C26	117.48 (14)
C22—C1—H1	109.2	N21—C22—C23	122.29 (13)
C1—O1—H1A	105.9 (15)	N21—C22—C1	116.07 (13)
C16—C11—C12	118.40 (15)	C23—C22—C1	121.63 (13)
C16—C11—C1	121.82 (14)	C24—C23—C22	119.09 (16)
C12—C11—C1	119.78 (14)	C24—C23—H23	120.5
C13—C12—C11	121.32 (16)	C22—C23—H23	120.5
C13—C12—H12	119.3	C25—C24—C23	119.03 (17)
C11—C12—H12	119.3	C25—C24—H24	120.5
C14—C13—C12	119.13 (17)	C23—C24—H24	120.5
C14—C13—H13	120.4	C24—C25—C26	118.56 (15)
C12—C13—H13	120.4	C24—C25—H25	120.7
C13—C14—C15	120.73 (17)	C26—C25—H25	120.7
C13—C14—Cl14	119.54 (15)	N21—C26—C25	123.55 (16)
C15—C14—Cl14	119.73 (15)	N21—C26—H26	118.2
C14—C15—C16	119.40 (18)	C25—C26—H26	118.2
C14—C15—H15	120.3		
			
O1—C1—C11—C16	129.16 (15)	C26—N21—C22—C23	1.2 (2)
C22—C1—C11—C16	−109.13 (16)	C26—N21—C22—C1	−177.31 (14)
O1—C1—C11—C12	−51.14 (17)	O1—C1—C22—N21	−156.41 (13)
C22—C1—C11—C12	70.57 (17)	C11—C1—C22—N21	84.12 (16)
C16—C11—C12—C13	0.9 (2)	O1—C1—C22—C23	25.1 (2)
C1—C11—C12—C13	−178.84 (14)	C11—C1—C22—C23	−94.42 (16)
C11—C12—C13—C14	0.2 (3)	N21—C22—C23—C24	−1.0 (2)
C12—C13—C14—C15	−1.3 (3)	C1—C22—C23—C24	177.48 (15)
C12—C13—C14—Cl14	178.90 (13)	C22—C23—C24—C25	0.3 (3)
C13—C14—C15—C16	1.4 (3)	C23—C24—C25—C26	0.0 (3)
Cl14—C14—C15—C16	−178.83 (15)	C22—N21—C26—C25	−0.9 (3)
C12—C11—C16—C15	−0.8 (3)	C24—C25—C26—N21	0.3 (3)
C1—C11—C16—C15	178.90 (16)	C11—C1—O1—H1A	−180.0 (17)
C14—C15—C16—C11	−0.3 (3)		

### Hydrogen-bond geometry (Å, º)

**Table:**

*D*—H···*A*	*D*—H	H···*A*	*D*···*A*	*D*—H···*A*
O1—H1*A*···N21^i^	0.84 (2)	2.01 (2)	2.8444 (18)	176 (2)

Symmetry code: (i) *x*, −*y*+1/2, *z*+1/2.
